# *Vibrio vulnificus* iron transport mutant has normal pathogenicity in *C. elegans*

**DOI:** 10.17912/micropub.biology.000124

**Published:** 2019-08-08

**Authors:** Adria K Bowles, David J Wynne, Ryan J Kenton

**Affiliations:** 1 University of Portland, Swindells Hall 108, Portland, Oregon USA 97203-5798

**Figure 1:**
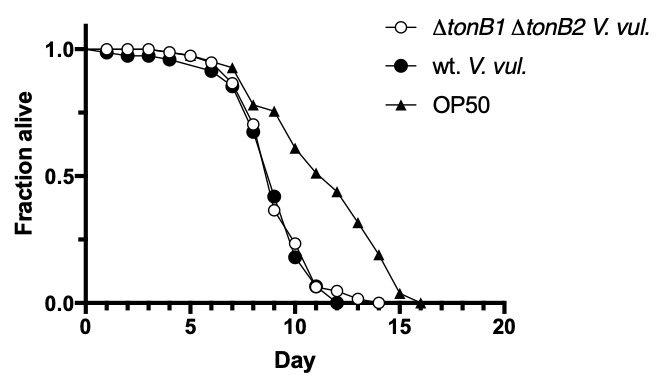
*fem-3(e2006)* L4 hermaphrodites were placed on lawns of OP50 *E. coli* (n=37), wildtype (wt.) *V. vulnificus* (n=65), or ∆*tonB1* ∆*tonB2* double mutant *V. vulnificus* (n=71) and assessed every 24 hrs. for survival. A Log-rank test indicated that there was no significant difference between survival on the two *V. vulnificus* strains (p=0.6), while both *V. vulnificus* strains cause significant reductions in survival relative to growth on OP50 (p<0.001).

## Description

*Vibrio vulnificus* is a gram-negative bacterium that is pathogenic to humans and capable of causing wound infections and primary septicemia (Gulig et al. 2005). Growth of *C. elegans* on pathogenic bacteria reduces their lifespan in a manner that recapitulates some aspects of the natural pathogenicity of many disease agents (for review, see Aballay and Ausubel 2002). *C. elegans* grown on *V. vulnificus* have reduced lifespans and this pathogenicity is diminished when worms are grown on *V. vulnificus* mutant strains defective in known virulence factors (Dhakal et al. 2006). We set out to use this host-parasite model to better understand the role of iron transport systems in *V. vulnificus* pathogenicity. Normal iron transport is required for full pathogenicity in mice due to the typically iron-limiting conditions in the host environment. *V. vulnificus* has three paralogs of the TonB iron transport system, known as the TonB1, TonB2, and TonB3 systems, and strains with deletion mutations in *tonB1* and *tonB2* (Δ*tonB1* Δ*tonB2*) are defective in iron transport (Kustusch et al. 2012). We tested whether this double mutant *V. vulnificus* strain would have reduced pathogenicity in *C. elegans*, as it does in mice. We confirmed that *C. elegans* grown on wildtype *V. vulnificus* reduced the median lifespan of animals from 12 days to 9 days. However, animals grown on the Δ*tonB1* Δ*tonB2* strain also had a median lifespan of 9 days and there was no statistically significant increase in survival of worms grown on the mutant strain (Fig. 1). It is possible that the iron transport systems were not essential for pathogenicity in these experiments because there was sufficient residual iron present despite the use of iron-limited CM9 plates. Further experiments with iron chelators introduced into the media are required to clarify whether the lack of dependence on iron transport is due to residual iron or a result of physiological differences between *V. vulnificus* infection of *C. elegans* intestine and its infection of the bloodstream of mice.

## Methods

Overnight broth cultures of each *V. vulnificus* strain were normalized to an OD_600_ of 0.3, spread onto CM9 plates, and incubated for 24 hours at 35°C. OP50 experiments were done on standard MYOB plates. For each replicate, 10 L4 hermaphrodites were transferred onto each plate on day 0. *fem-3* animals at the restrictive temperature of 25°C, which have been shown to have normal lifespan (Kenyon et al. 1993), were used so animals did not have to be transferred away from progeny. Plates incubated at 25°C were scored every 24hr for the number of remaining live worms. A worm was considered dead when it no longer responded to touch with a pick. Data was graphed using Prism software (Graphpad), and statistical significance was determined using the Log-Rank test with the assumption of proportional hazards maintained.

## Reagents

CM9 plates

1.5% agar, 1x M9 salts (60 g Na2HPO4, 30 g KH2PO4, 50 g NaCl, 10 g NH4Cl per liter [pH 7.2]), 0.2% Casamino Acids, 0.5% glucose, 10 μM CaCl2, 100 μM MgSO4, 100 µg/ml Ampicillin.

MYOB plates

2% agar, 0.55g Tris-Cl, 0.24g Tris-OH, 3.1g Peptone, 8mg cholesterol, 2gNaCl per liter.

Strains

**Table:** 

Strain	Genotype	Availability
CB3844	*fem-3(e2006)* IV	Available from the CGC

V. vulnificus Strains

**Table:** 

Strain	Genotype	Availability
CMCP6	wild type	R. J. Kenton
AA-9	*ΔtonB1* *ΔtonB2*	R. J. Kenton
